# Lesion durability found during mandated percutaneous catheter ablation after surgical cryo-ablation for treatment of non-paroxysmal atrial fibrillation

**DOI:** 10.1186/s13019-024-02889-3

**Published:** 2024-06-27

**Authors:** Alan Bulava, Aleš Mokráček, Petr Němec, Dan Wichterle, Pavel Osmančík, Petr Budera, Petr Kačer, Linda Vetešková, Tomáš Skála, Petr Šantavý, Jan Chovančík, Piotr Branny, Vitalii Rizov, Miroslav Kolesár, Iva Šafaříková, Marian Rybář, SURHYB Trial Investigators

**Affiliations:** 1 Faculty of Health and Social Sciences, University of South Bohemia in České Budějovice and Cardiac Centre, České Budějovice Hospital, České Budějovice, Czechia; 2 Centre of Cardiovascular Surgery and Transplantation and Faculty of Medicine, Masaryk University, Brno, Czechia; 3 Institute for Clinical and Experimental Medicine, Prague, Czechia; 4 3rd Faculty of Medicine, Charles University and University Hospital Královské Vinohrady, Prague, Czechia; 5 Faculty of Medicine and Dentistry, Palacký University and University Hospital Olomouc, Olomouc, Czechia; 6 Hospital Agel Třinec - Podlesí, Třinec, Czechia; 7 Masaryk Hospital, Ústí Nad Labem, Czechia; 8 Faculty of Biomedical Engineering, Czech Technical University in Prague, Kladno, Czechia

**Keywords:** Concomitant atrial fibrillation ablation, Staged hybrid ablation, CryoMaze procedure, Electrical conduction, Electrophysiological study, Gaps localization

## Abstract

**Objectives:**

Current recommendations support surgical treatment of atrial fibrillation (AF) in patients indicated for cardiac surgery. These procedures are referred to as concomitant and may be carried out using radiofrequency energy or cryo-ablation. This study aimed to assess the electrophysiological findings in patients undergoing concomitant cryo-ablation.

**Methods:**

Patients with non-paroxysmal AF undergoing coronary artery bypass grafting and/or valve repair/replacement were included in the trial if concomitant cryo-ablation was part of the treatment plan according to current guidelines. The patients reported in this study were assigned to undergo staged percutaneous radiofrequency catheter ablation (PRFCA), i.e., hybrid treatment, as a part of the SURHYB trial protocol.

**Results:**

We analyzed 103 patients who underwent PRFCA 105 ± 35 days after surgery. Left and right pulmonary veins (PVs) were found isolated in 65 (63.1%) and 63 (61.2%) patients, respectively. The LA posterior wall isolation and mitral isthmus conduction block were found in 38 (36.9%) and 18 (20.0%) patients, respectively. Electrical reconnections (gaps) in the left PVs were more often localized superiorly than inferiorly (57.9% vs. 26.3%, *P* = 0.005) and anteriorly than posteriorly (65.8% vs. 31.6%, *P* = 0.003). Gaps in the right PVs were more equally distributed anteroposteriorly but dominated in superior segments (72.5% vs. 40.0%, *P* = 0.003). There was a higher number of gaps on the roof line compared to the inferior line (131 (67.2%) vs. 67 (42.2%), *P* < 0.001). Compared to epicardial cryo-ablation, endocardial was more effective in creating PVs and LA posterior wall isolation (*P* < 0.05). Cryo-ablation using nitrous oxide (N_2_0) or argon (Ar) gas as cooling agents was similarly effective (*P* = NS).

**Conclusions:**

The effectiveness of surgical cryo-ablation in achieving transmural and durable lesions in the left atrium is surprisingly low. Gaps are located predominantly in the superior and anterior portions of the PVs and on the roof line. Endocardial cryo-ablation is more effective than epicardial ablation, irrespective of the cooling agent used.

## Introduction

The prevalence of atrial fibrillation (AF) in patients indicated for cardiac surgery is higher than in the general population [1, 2]. The Cox MAZE IV is the gold standard for surgical treatment of AF [3, 4]. Currently, the procedure is performed using either radiofrequency (RF) energy or cryo-thermal tissue destruction (CryoMaze) [5]. Rather than stand-alone operations, these procedures are more often performed as concomitant operations following coronary artery bypass or valve surgery.

The efficacy of the concomitant CryoMaze as a treatment for persistent AF has been demonstrated in several studies, but inconsistent extent of CryoMaze and intensity of rhythm monitoring after the procedure has led to discordant “freedom from AF” rates reported between 47–and 95% [6, 7]. Moreover, incomplete lines after CryoMaze are not infrequent [8, 9], and the recurrence of electrical conduction through cryo-lesions could be pro-arrhythmic [10, 11]. As a result, patients with recurrent symptomatic atrial arrhythmias after CryoMaze are often referred for the electrophysiology examination, during which the surgical lines can be mapped and completed by touch-up percutaneous RF catheter ablation (PRFCA).

As achieving the permanent lesion set in the left (and right) atrium is of paramount importance for the elimination of recurring AF or atrial tachycardias (AT), our trial aimed at systematic exploration of the effectiveness of cryo-ablation in creating durable pulmonary vein (PV) isolation and linear lines in the left atrium (LA). The data on the actual transmurality and durability of surgically created cryo-lesions could provide valuable feedback for the surgeons, should the location of the sites of electrical reconnections (hereafter referred to as “gaps”) in the circumferential or linear lines be predominantly clustered in certain positions. The presented study is a substudy of the Sequential HYBrid Ablation versus SURgical CryoMaze Alone for Treatment of Atrial Fibrillation Trial (SURHYB Trial).

## Methods

### Ethical statement

The SURHYB Trial was conducted as an investigator-initiated, multicenter, open-label, parallel-group, randomized controlled trial in seven major complex cardiovascular centers in Czechia. The details of the trial design and primary results have been published [12, 13]. The trial protocol was approved by the institutional ethics committees at all participating institutions. The trial followed the Helsinki Declaration of 1964, its later amendments, and the Good Clinical Practice Guidelines. Written informed consent was obtained from all patients before enrolment, and the recruitment period lasted between May 1, 2019, and March 31, 2022. The trial was registered in the Czech Clinical Trials Registry, cz-020420181253 (accessible at www.ablace.cz).

### Patients

Patients ≥ 18 years with non-paroxysmal AF who were indicated for cardiac surgery (coronary artery bypass grafting, valve surgery, or a combination of both) were screened. They were eligible for the trial if suitable for the concomitant CryoMaze procedure based on expert consensus statements [14]. Exclusion criteria comprised AF secondary to a reversible cause, LA diameter (in parasternal long axis view) > 55 mm, previous surgical or catheter ablation for AF/AT, chronic kidney disease (stage ≥ 4), contraindication to systemic anticoagulation, estimated life expectancy < 1 year, and inability to mentally/physically comply with all trial requirements.

### Cryo-ablation procedure

**Fig. 1:**
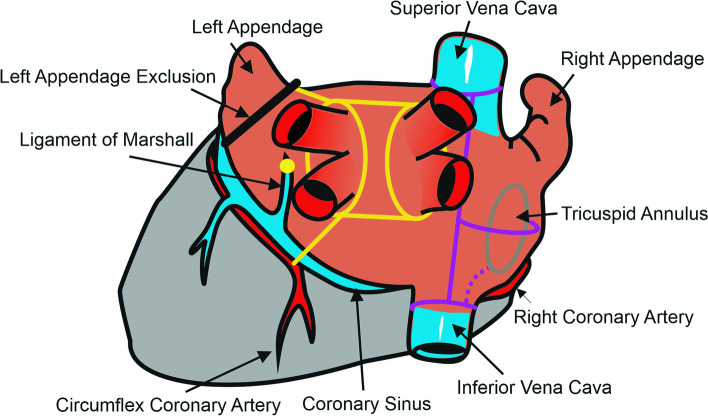
Scheme of surgical cryo-lesions. The CryoMaze procedure consisted of mandatory circular lesions (yellow color) around the ipsilateral right and left pulmonary veins with linear lesions toward the auricle and between the superior and inferior pulmonary veins to isolate the LA posterior wall. A mitral isthmus ablation line was created in all patients from the inferior connecting lesion towards the mitral annulus. In addition, Marshall's ligament was cut off (yellow dot), and left atrial appendage exclusion was performed (black line) using an AtriClip device, staplers, or cut-and-sew technique. Right atrial lesions (purple) were performed at the surgeon's discretion. Such lesions may have included but were not limited to superior/inferior vena cava isolation, intercaval lesion, lateral line connecting intercaval lesion to the tricuspid annulus, and cavotricuspid isthmus lesion (dotted purple line)

The cryo-ablation was carried out using nitrous oxide (N_2_0) with the aluminum cryoICE ablation probe (AtriCure, Inc., Cincinnati, Ohio, United States) or the argon (Ar) with the stainless steel Cardioblate CryoFlexTM 10-S probe (Medtronic, Inc., Minneapolis, Minnesota, United States). The protocol consisted of mandatory circular lesions around the ipsilateral right and left PVs with linear lesions between the superior and inferior contralateral PVs to isolate the LA posterior wall (box lesion). A mitral isthmus ablation line was created in all patients from the inferior connecting lesion towards the mitral annulus. Whenever possible, the isthmus line was done both epicardially and endocardially. All cryo-lesions were endocardial in cases of mitral valve surgery, i.e., when the LA was cut open. In the remaining indications for cardiac surgery, cryo-lesions were applied epicardially on-pump but with the heart still beating. Surgeons were mandated to perform at least one freezing lasting for at least two minutes for each lesion with clearly overlapping regions when linear lesions touched circumferential lesions around PVs under visual control. In addition, the ligament of Marshall was cut off in all patients. The LA appendage (LAA) was excluded in patients with a CHA_2_DS_2_-VASc score ≥ 2, and a line into the LAA from the left superior PV was extended in such cases. The right atrium (RA) cryo-lesions were performed at the surgeon's preference. Such lesions may have included but were not limited to superior/inferior vena cava isolation, intercaval lesion, and cavotricuspid isthmus (CTI) lesion (Fig. 1).

Patients were randomly assigned in a 1:1 ratio to (i) the Hybrid Group or (ii) the Surgery Group. Randomization was performed post-operatively, which ensured that the surgeons performing cryo-ablation were unaware of treatment group allocation during surgery. Patients randomized to the Hybrid Group were admitted for a staged PRFCA 90 ± 20 days after the surgical procedure. Dense electroanatomic mapping of the LA and RA was performed using a CARTO3 navigation system and a Thermocool SmartTouch® ablation catheter (Biosense Webster, Inc., USA) in combination with multipolar circular mapping catheter (Lasso™, Biosense Webster, Inc., USA) to provide information about the location of the cryo-lesions.

**Fig. 2:**
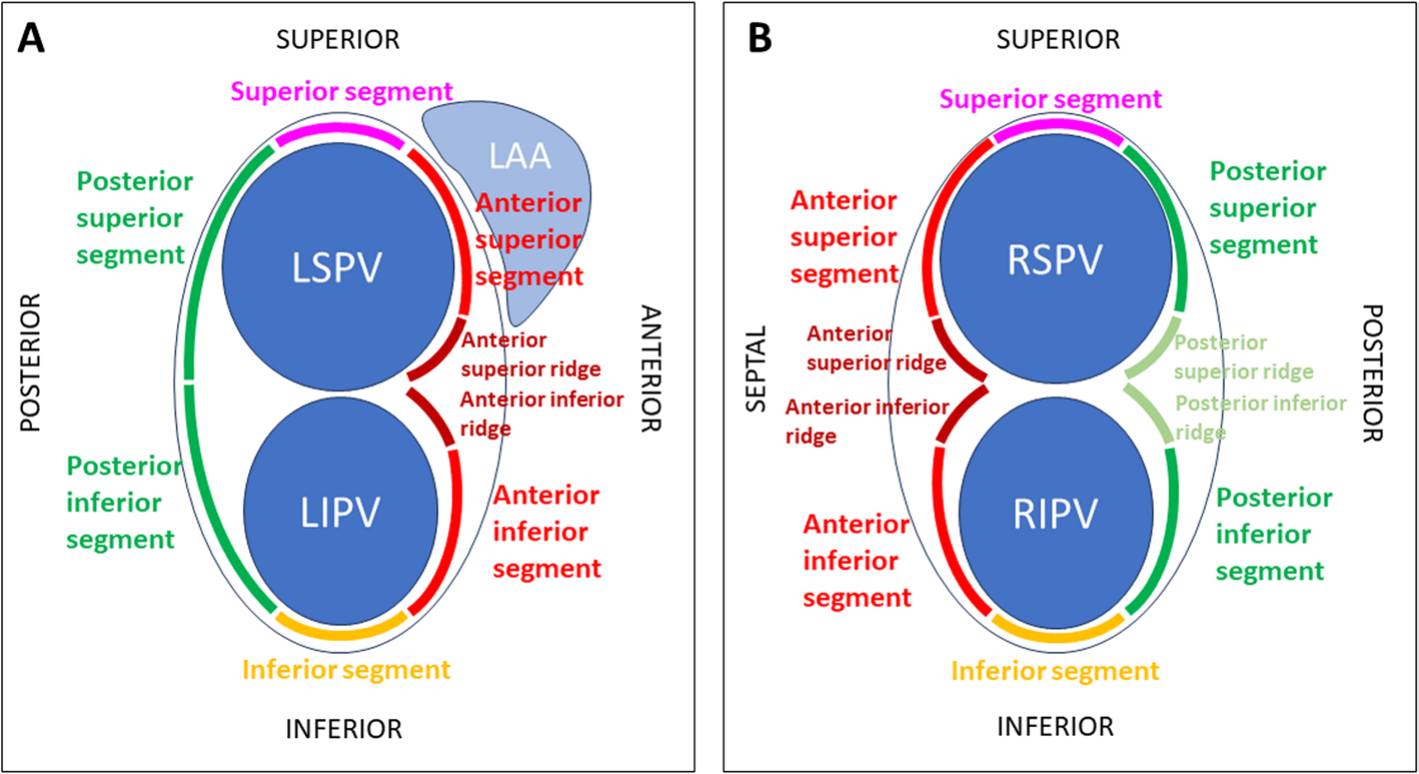
For evaluation of electrical reconnections (gaps), the antrum of the left pulmonary veins (**A**) was schematically divided into one superior and inferior segment and two posterior and four anterior segments (adjacent to the respective pulmonary vein). Similarly, the antrum of the right pulmonary veins (**B**) was divided into superior and inferior segments and four anterior and four posterior segments. LAA, left atrial appendage; LIPV, left inferior pulmonary vein; LSPV, left superior pulmonary vein; RIPV, right inferior pulmonary vein; RSPV, right superior pulmonary vein

The circumference of the left and right PVs was divided into eight and ten segments, respectively (Fig. 2). Entry and exit block in sinus rhythm and during pacing maneuvers using a multipolar circular mapping catheter placed in the PVs was meticulously carried out to record the localization of the gap(s) on the PV circumference. Similarly, the roof line (RL) and inferior connecting line (IL) were divided into thirds: RL_1_ and IL_1_ adjacent to the left PVs, RL_2_ and IL_2_ in the middle, and RL_3_ and IL_3_ adjacent to the right PVs. If potentials were found on the LA posterior wall using a multielectrode circular catheter during sinus rhythm, the earliest electrical activation was searched during LA posterior wall pacing to identify the place of conduction anterior to the roof line or inferior to the inferior line respectively. The goal was to close the gaps in all circular and linear lines using RF energy and touch-up ablation and, in the RA, create a bidirectional conduction block on the CTI. Finally, all procedural ATs, spontaneous or induced, were mapped and ablated. For induction, incremental atrial pacing up to 300 bpm was used. No pharmacological challenge was used to facilitate the induction of AF or AT. Participants in the Surgery Group (control group) received surgical cryo-ablation only, and no data on PV isolation or completeness of linear lesions are available for this group.

### Statistical analysis

Continuous variables are reported as means with standard deviation (SD) or medians with interquartile range (IQR) and compared between the trial groups by a Student´s t-test for independent samples or Mann–Whitney test, as appropriate according to the normality of data distribution. Categorical variables are reported as frequencies and proportions and compared between the trial groups by the chi-squared test. The null hypothesis (no difference between predefined comparisons) was rejected by a 2-sided test at the significance level of 0.05. We conducted the statistics using software R, version 4.3.1.

## Results

A total of 236 patients were enrolled at seven sites. Due to early postoperative death in one patient or informed consent withdrawal (four patients), 115 and 116 patients were finally assigned to the Hybrid and Surgery Groups, respectively. In the Hybrid Group, two patients withdrew their consent to the trial before the scheduled PRFCA, and three died before the planned PRFCA. Seven patients from the Hybrid Group ultimately refused to undergo PRFCA, so we were able to analyze data only from 103 patients in the Hybrid Group concerning the effectiveness of the cryo-ablation procedure, which forms the basis of this report.

Baseline demographic and clinical characteristics are listed in Table 1. The mean continuous AF duration before inclusion in the trial was 2.3 ± 0.8 years. Current or prior ineffective use of Class IC or Class III antiarrhythmic drugs (AADs) was documented in 90% of patients. Procedural aspects of the CryoMaze and PRFCA procedures are shown in Tables 2 and 3, respectively.

**Table 1:** Baseline clinical characteristics of the patients assigned to undergo endocardial mapping and catheter ablation procedure

	**Hybrid Group *n* = 113**^a^
Age (years)	68.5 ± 7.2
Male	80 (70.8)
Body mass index (kg/m^2^)	30.8 ± 4.9
Persistent atrial fibrillation	57 (50.4)
Long-standing atrial fibrillation	56 (49.6)
Congestive heart failure	90 (79.6)
NYHA Class	
I	10 (8.8)
II	44 (38.9)
III	34 (30.1)
IV	2 (1.8)
CHA_2_DS_2_-VASc score	
0 – 2	19 (16.8)
3 – 5	78 (69.0)
6 – 9	16 (14.2)
Left atrium diameter (cm)	4.8 ± 0.5
Left ventricular ejection fraction (%)	56.9 ± 11.5
History of electrical cardioversion	48 (42.5)
Arterial hypertension	96 (85.0)
Diabetes mellitus	40 (35.4)
Coronary artery disease	49 (43.4)
Transient ischemic attack / Stroke	14 (12.4)

Values are the number (percentage) of patients or mean ± standard deviation

^a^Baseline data are presented for the whole population of the Hybrid Group; however, 12 patients were excluded for the final efficacy analysis (3 deaths, 2 consent withdrawals, and 7 patients who ultimately refused to undergo the endocardial mapping and catheter ablation)

**Table 2:** Cardiac surgery characteristics of the patients assigned to undergo endocardial mapping and catheter ablation procedure

	**Hybrid Group (*n* = 113)**^a^
Procedural time (min)	223 ± 60
Cardiopulmonary bypass time (min)	121 ± 41
Aortic clamp time (min)	86 ± 36
Hospitalization length (days)	13.6 ± 7.5
**Type of procedure**	
Coronary artery bypass grafting	50 (44.2)
Number of bypass grafts	2.2 ± 1.0
Complete revascularisation^b^	42 (84.0)
Mitral valve repair	28 (24.8)
Mitral valve replacement	15 (13.3)
Tricuspid valve repair	25 (22.1)
Aortic valve replacement	40 (35.4)
**Type of cryo-energy**	
Argon-based cryoablation	23 (20.4)
Nitrous oxide-based cryoablation	90 (79.6)
**CryoMaze ablation details**	
Left pulmonary veins ablated	113 (100.0)
Right pulmonary veins ablated	113 (100.0)
Left atrial appendage occlusion	101 (89.4)
AtriClip device	69 (61.1)
Staplers	0 (0)
Cut-and-sew technique	32 (28.3)
Line to left atrial appendage	101 (89.4)
Left atrial box lesion created	113 (100.0)
Mitral isthmus line created	97 (85.8)
Marshall ligament cut-off	81 (71.7)
Superior vena cava ablated	13 (11.5)
Inferior vena cava ablated	0 (0.0)
Intercaval line created	45 (39.8)
Cavotricuspid isthmus line created	8 (7.1)
Other lines in the right atrium	19 (16.8)
Other lines in the left atrium	0 (0.0)

Values are the number (percentage) of patients or mean ± standard deviation

^a^Cardiac surgery data are presented for the whole population of the Hybrid Group; however, 12 patients were excluded for the final efficacy analysis (3 deaths, 2 consent withdrawals, and 7 patients who ultimately refused to undergo the endocardial mapping and catheter ablation)

^b^Percentage of complete revascularisation in the subgroup of patients undergoing bypass surgery

**Table 3:** Characteristics of the catheter ablation procedure

Ablation Procedure Data (*n* = 103)	
Time from CryoMaze to catheter ablation (days)	105 ± 35
Procedural time (min)	138 ± 52
Radiofrequency energy application time (min)	27.0 ± 16.3
Fluoroscopy time (min)	6.9 ± 3.2
Fluoroscopy dose (mGy.cm^2^)	6507 ± 7300
Hospitalization length (days)	2.6 ± 1.2
**Ablation procedure details**	
Patients presented with sinus rhythm	74 (71.8)
Ablation catheter with contact force sensors	81 (78.6)
Ablation catheter without contact force sensors	22 (21.4)

Values are the number (percentage) of patients or mean ± standard deviation

### Effectiveness of surgical cryo-ablation

An electrophysiological procedure was performed 105 ± 35 days after the concomitant cryo-ablation procedure. Seventy-five of 103 patients (72.8%) presented with SR at the beginning of the PRFCA procedure. Nine patients (8.7%) had AF. In comparison, 13 (12.6%) had regular ATs: two patients presented with focal RA tachycardias and 11 patients with macroreentry LA tachycardias (roof-dependent in three, perimitral in seven, and AT using the conduction gap between the right PVs in one patient). Typical RA flutter was present in 6 patients (5.8%).

**Fig. 3:**
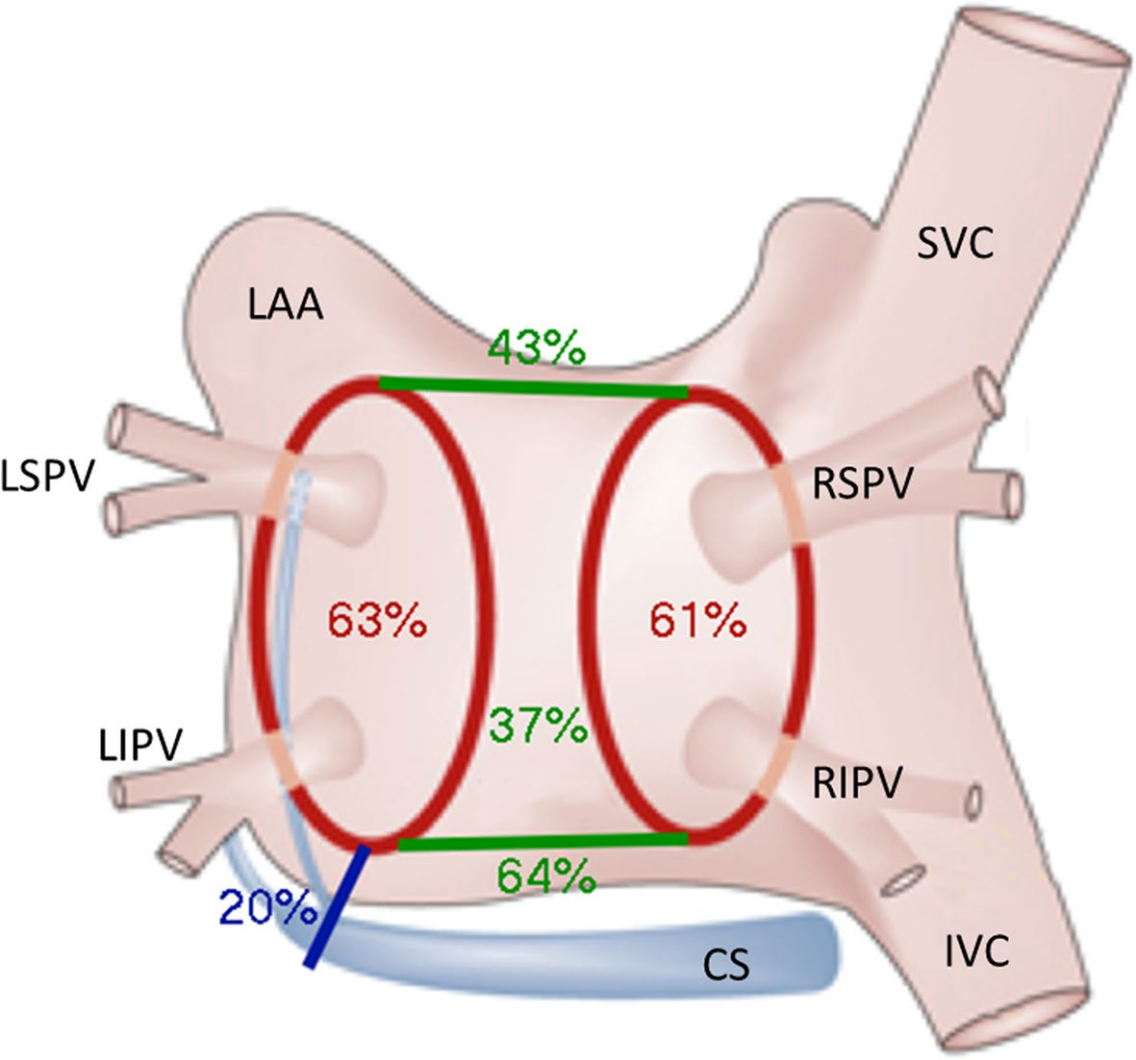
Completeness of cryo-thermal ablation lines after the CryoMaze procedure. Circumferential lines around pulmonary veins (red), box lesion (green), and mitral isthmus line (blue). Percentages indicate the success of electrical isolation and proven bidirectional conduction block found during electrophysiological examination three months after the surgical cryo-ablation. CS, coronary sinus; IVC, inferior vena cava; LAA, left atrial appendage; LIPV, left inferior pulmonary vein; LSPV, left superior pulmonary vein; RIPV, right inferior pulmonary vein; RSPV, right superior pulmonary vein; SVC, superior vena cava

PV isolation and box lesion were attempted during cardiac surgery in all 103 study group patients. Left and right PVs were confirmed to be isolated in 65 (63.1%) and 63 (61.2%) patients, respectively, after concomitant cryo-ablation. All PVs were durably isolated in 49 patients (47.6%). The RL between the superior PVs was complete in 44 cases (42.7%), and the IL between the inferior PVs was successfully ablated in 66 patients (64.1%). Thus, the complete box lesion was found in 38 (36.9%) patients after cardiac surgery prior to PRFCA (Fig. 3).

Cryo-ablation of the mitral isthmus line was carried out in only 90 patients, mainly due to technical reasons accompanying surgical procedure. The bidirectional conduction block endured in 18 of them (20.0%). The complete LA lesion set (i.e., PV isolation complemented by the box lesion and mitral isthmus block) was achieved in 19 patients (18.4%) after the LA cryo-ablation procedure, as evidenced by the percutaneous catheter procedure. After percutaneous endocardial touch-up RF ablation, the PV isolation was completed in all patients (100%), box lesion in 92 patients (89.3%), and mitral isthmus block in 96 patients (93.2%).

### Localizations of electrical reconnections

**Fig. 4:**
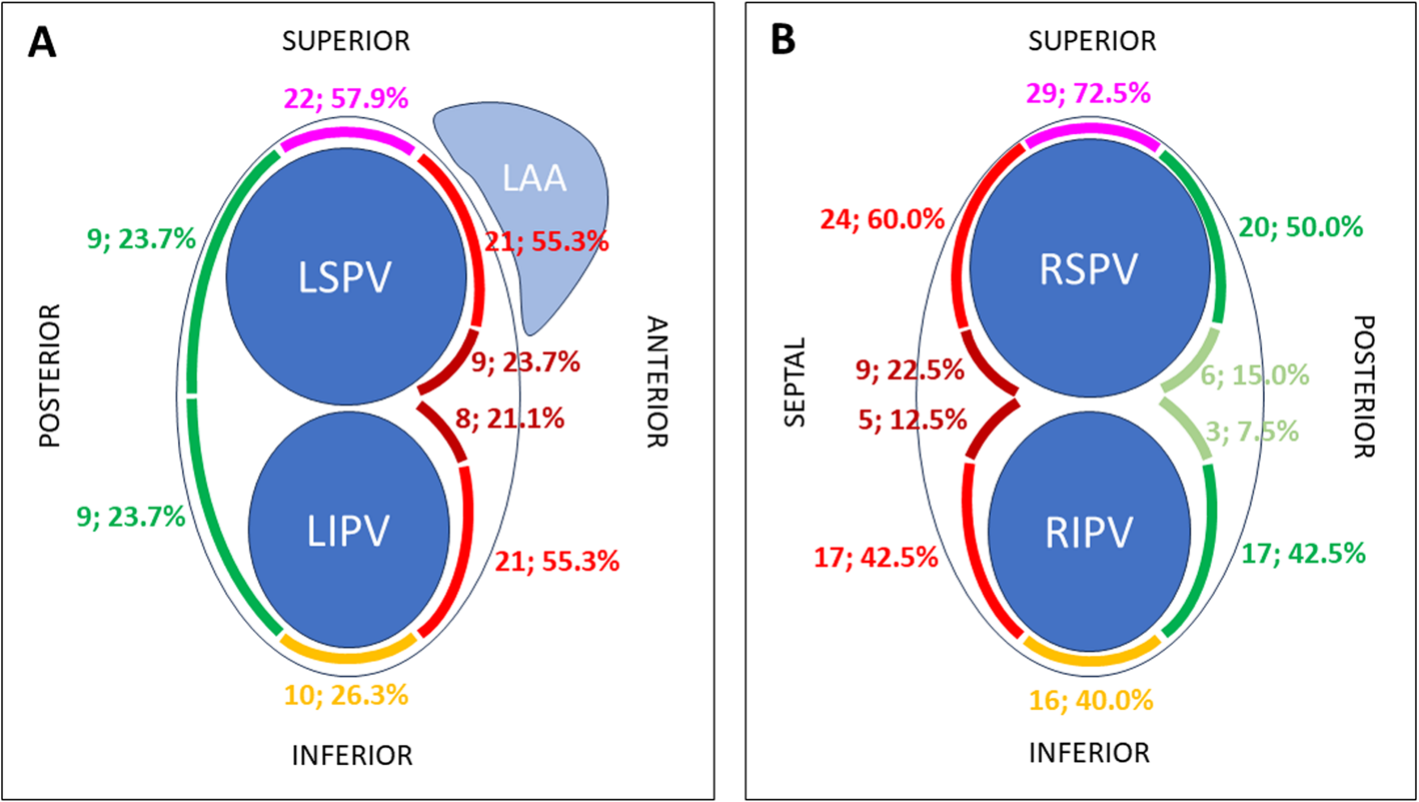
Absolute numbers indicate the number of electrical reconnections (gaps) in the respective region (see Fig. 2 for reference). Percentages indicate the relative proportion of gap localization in the area concerning the total number of evaluated anterior, posterior, superior, and inferior segments. LAA, left atrial appendage; LIPV, left inferior pulmonary vein; LSPV, left superior pulmonary vein; RIPV, right inferior pulmonary vein; RSPV, right superior pulmonary vein

Left PVs were not surgically isolated in 38 patients, providing 38 PV antral segments in the superior and inferior portions of the left PV circumference for gap analysis. As anterior and posterior parts of the left PVs were divided into four and two segments, respectively, 152 and 76 anterior and posterior segments were evaluated for gaps. Most conduction gaps were located anteriorly and superiorly (Fig. 4). Gaps were found in 59 of 152 (38.8%) anterior segments compared to 18 of 76 (23.7%) posterior segments (*P* = 0.022). Gaps were also more often localized superiorly than inferiorly (22 (57.9%) vs. 10 (26.3%) segments, *P* = 0.005).

Right PVs were not surgically isolated in 40 patients. Therefore, 40, 160, 40, and 160 segments in the superior, anterior, inferior, and posterior PV antra were available for gap analysis. No significant difference was found comparing the number of gaps anteriorly and posteriorly (55 (34.4%) vs. 46 (28.8%) segments, *P* = 0.28). Gaps were more often localized superiorly than inferiorly (29 (72.5%) vs. 16 (40.0%) segments, *P* = 0.003).

Regarding the box lesion, electrical reconnection of the LA posterior wall was identified in 65 patients. Localization of the gaps on the roof line did not differ (42 (64.6%), 45 (69.2%), and 44 (67.7%) gaps on RL_1_, RL_2,_ and RL_3_, respectively, *P* = 0.849); neither did the localization of gaps on the inferior connecting line: 18 (27.7%), 20 (30.8%) and 29 (44.6%) gaps on IL_1_, IL_2_ and IL_3_, respectively, *P* = 0.096). Altogether, there was a significantly higher number of gaps on the RL compared to IL (131 (67.2%) vs. 67 (42.2%), *P* < 0.001).

### Factors influencing effectiveness

**Fig. 5:**
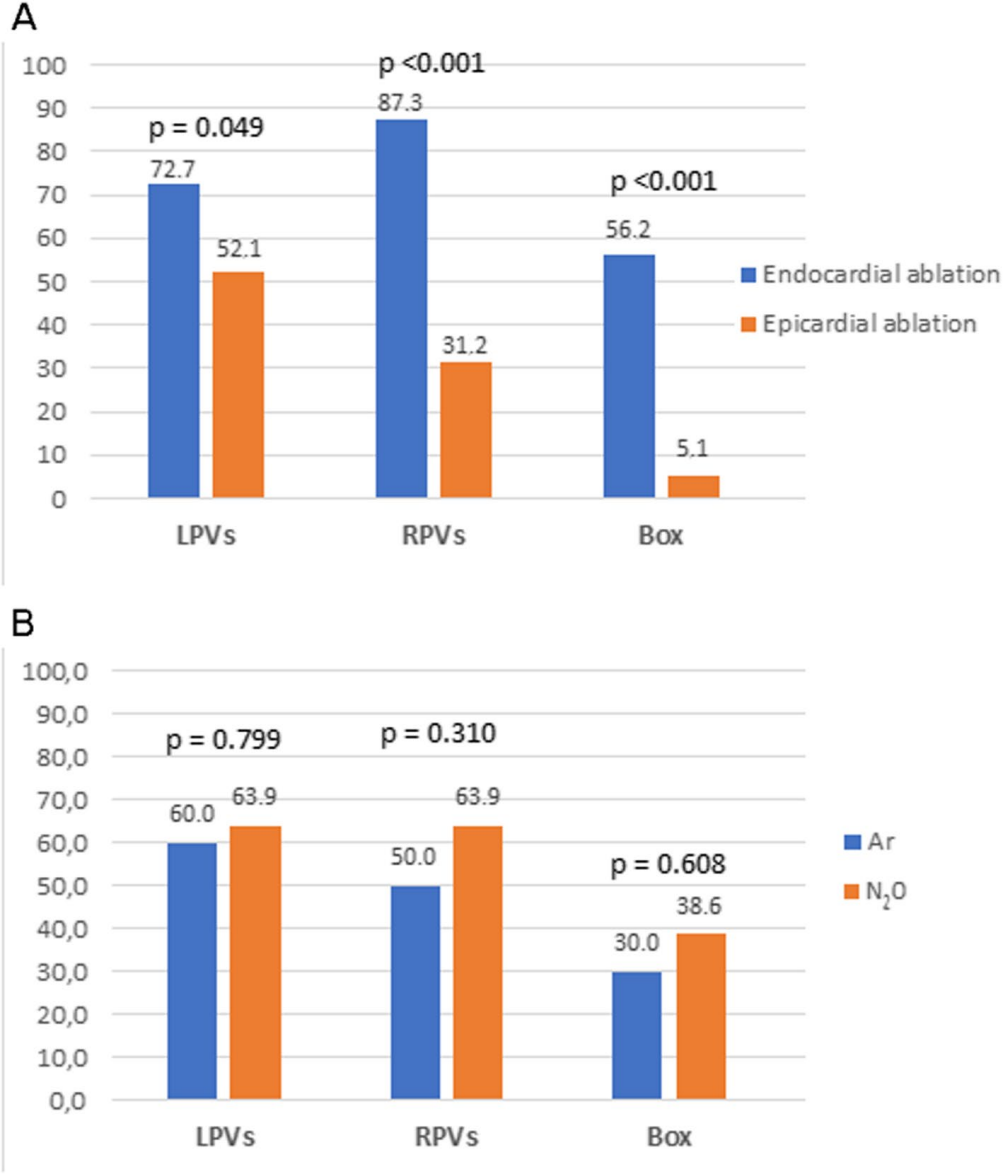
Comparison of the successful cryo-lesions (in percentages) depending on the epicardial vs. endocardial application of the cryo-energy (**A**) and the type of cryo-energy used (**B**). Ar, argon; Box, box lesion, i.e., left atrial posterior wall isolation; LPVS, left pulmonary veins; N_2_0, nitrous oxide; RPVs, right pulmonary veins

PVs were ablated from the endocardium in 55 (53.4%) patients, while 48 (46.6%) patients were ablated epicardially. Box lesions were created endocardially in 64 (62.1%) patients and epicardially in 39 (37.9%). A significantly higher proportion of the PVs was isolated after endocardial vs. epicardial cryo-ablation (72.7% vs. 52.1%, *P* = 0.049 for the left PVs, and 87.3% vs. 31.2%, *P* < 0.001 for the right PVs). Similarly, endocardial cryo-ablation was more effective in achieving posterior LA wall isolation than epicardial cryo-ablation (56.2% vs 5.1%, *P* < 0.001, Fig. 5A).

Ar-based cryo-ablation was used in 20 (19.4%) patients, while the remaining 83 (80.6%) patients underwent N_2_0-based cryo-ablation. No significant differences were noted when comparing the two media for deep freezing (Fig. 5B).

## Discussion

The CryoMaze procedure is deemed an effective therapeutic option for patients with AF during concomitant valve or bypass surgery. According to available US registries, cryo-energy is used in approximately 30–50% of patients undergoing concomitant surgical ablation, primarily from the epicardial approach [15]. However, only limited data exist on the effectiveness of surgically created cryo-lesions. Our multicenter trial aimed to systematically describe electrophysiological findings in all patients randomized to the hybrid treatment strategy irrespective of clinical efficacy, i.e., AF/AT recurrences. The main results are as follows: (1) Only less than half of the patients have all PVs isolated after surgical cryo-ablation, (2) approximately 60% of patients with surgically attempted box lesions have posterior LA wall reconnected, (3) complete durable LA lesion set was accomplished in less than one-fifth of patients, (4) epicardial cryo-ablation is way less effective than endocardial, and (5) Ar-based cryo-ablation is comparable with N_2_0.

Current evidence-based recommendations support surgical ablation in patients with AF undergoing cardiac surgery for other indications [14, 16]. According to the 2017 guidelines of the Society of Thoracic Surgeons [17], surgical ablation is highly recommended as a Class IA procedure to restore sinus rhythm along with mitral valve surgery. It is also recommended as a Class IB procedure in conjunction with coronary artery bypass or aortic valve replacement. Therefore, the contemporary utilization of surgical AF ablation has increased across all operative categories. As the performance of surgical AF ablation is accompanied by a reduction in mortality and stroke [15], most likely due to the conversion of AF into normal sinus rhythm [18] and LAA occlusion [19], the surgeons should aim at the maximal effectiveness of the created cryo-lesions.

However, a more detailed analysis of the rhythm outcomes after surgical AF ablation brought tentative results. Depending on the intensity of rhythm monitoring, reports on concomitant CryoMaze procedures showed efficacy rates only between 47 and 76% [6, 7, 20]. Sinus rhythm maintenance depends on an appropriate durable lesion set [10, 11]. In our previous single-center study [8] in patients with non-paroxysmal AF, complete PV isolation and box lesions were present in only 66% and 51% of patients, respectively, in line with our current observation in a larger multicentric cohort. The importance of achieving a durable transmural lesion set may be demonstrated in studies reporting sequential hybrid AF treatment protocol results. Touch-up catheter ablation of the incomplete surgical lesions led to excellent efficacy results both in observational and randomized studies [21–23]. Two small observational studies on hybrid procedures after the concomitant CryoMaze reported 86% overall freedom from AF/AT at 12 months [8] or 73% after a 10-year follow-up [24]. The SURHYB trial [13], the first randomized trial on this topic, showed an impressive 62% relative risk reduction of recurrent AF/AT after the hybrid strategy (67.4% vs. 41.1% arrhythmia recurrence rate in the surgery-alone vs. hybrid treatment, respectively), using implantable ECG monitors.

In our study, we tried for the first time to systematically analyze the location of gaps based on prespecified protocol during electrophysiological study. Regarding the left PVs, we found that most conduction gaps were located anteriorly and superiorly. Similarly, more gaps were found in the superior aspect regarding the right PVs. The same applies to the roof line, where the LA wall thickness is significantly greater than the inferior LA portions.

In the past, several articles were published on cryo-energy, comparing epicardial and endocardial approaches [22, 25, 26]. However, these studies were performed under laboratory conditions or in an animal model. Our previous non-randomized single-center observation showed a higher prevalence of PV or LA posterior wall isolations when the cryo-energy was applied endocardially [9]. This multicenter study fully confirmed this. Epicardial cryo-ablation in our patient cohort led to isolation in only 50% of left PVs. The efficacy was even weaker in the right PVs, leaving more than two-thirds of the right PVs reconnected. Isolation of the LA posterior wall using epicardial ablation was extremely meager (only 5% of patients). Based on our results, the technique of epicardial cryo-ablation should be revisited or even wholly abandoned since we are perhaps left without any chances to improve the method of epicardial ablation using current tools and cryo-energy. The main reason is that even longer or repeated application of cryo-energy will likely make no significant difference in the outcome. Cryo-energy penetration through the epicardial fat to create a durable transmural lesion is seemingly poor, and this holds true even for RF energy [27]. Thus, at present, cryo-ablation of AF might be perhaps reserved only for patients with mitral (or tricuspid) valve surgery, in whom endocardial application of the cryo-energy is possible.

Available cryo-ablation devices use nitrous oxide (N_2_0) or argon (Ar) gas as cooling agents. One small single-center randomized study showed a similar 1-year sinus rhythm maintenance rate using N_2_O-based cryo-ablation vs. Ar-based cryo-ablation in patients with persistent AF [28]. Another randomized trial showed that both N_2_O- and Ar-based cryoprobes provide similar rates of sinus rhythm maintenance and freedom from major adverse cardiovascular and cerebrovascular events at the 5-year follow-up [29]. For the first time, our study provided the rationale for similar clinical outcomes when comparing N_2_0 and Ar. By these clinically focused studies, we could not demonstrate different PV or LA posterior wall isolation rates between the N_2_0-based and Ar-based cryo-ablation in our research.

The trial has limitations that need to be addressed. First, we studied only patients allocated to the Hybrid Group by the study design. Should both study groups undergo electrophysiology examination, more robust data might have been acquired. However, as the patients in our study were randomized, we consider more than a hundred patients a fairly representative cohort. Only centers with vast experience in AF cryo-ablation (i.e., more than 100 procedures per year) were invited to participate in this trial. Still, we cannot wholly exclude methodological variabilities between the centers or individual operators. Nevertheless, the multicenter nature of our study is likely to offer a clinically realistic picture of the contemporary results of cryo-ablation treatment of AF. Second, patients allocated to the Hybrid Group had to undergo a mandated electrophysiology examination regardless of arrhythmia symptoms or recurrence. The durability of the lesion set in mandated remapping studies is usually higher than in patients with arrhythmia recurrence. However, this effect might be less robust in our sicker population, where arrhythmia recurrence is less dependent on PV isolation only. Yet, even if such an effect might affect our study, the result of durable PV isolation and box lesion using surgical cryo-ablation might have been even worse in patients with clinically recurrent arrhythmias. Last but not least, the study was not powered to detect differences in gap locations or freezing technologies used during CryoMaze, so these results should still be interpreted cautiously.

## Conclusion

The actual effectiveness of cryo-ablation in achieving transmural and durable lesions in the LA is surprisingly low. Gaps are located predominantly in the superior and anterior portions of the PVs and on the left atrial roof line. Endocardial cryo-ablation is more effective than epicardial ablation, irrespective of the type of cooling agent. Proper training is needed for AF surgery, and the surgical community should take AF treatment more seriously and make it a specialty with dedicated surgeons as with other surgical procedures. Novel tools or techniques are mandatory to improve the results of concomitant surgical AF ablation.

## Data Availability

The datasets used and/or analyzed during the current study are available from the corresponding author upon reasonable request.
